# Supplementary material for: Scopolamine induced learning deficit in marmosets
Source: bioRxiv. 2025 Nov 18:2025.11.18.688868. Preprint. [Version 1] doi: 10.1101/2025.11.18.688868 (PMC12667990; doi:10.1101/2025.11.18.688868)
Supplement: Supplement 1 [file NIHPP2025.11.18.688868v1-supplement-1.pdf]

bioRxiv preprint doi: https://doi.org/10.1101/2025.11.18.688868; this version posted November 18, 2025. The copyright holder for this preprint (which was not certified by peer review) is the author/funder. This article is a US Government work. It is not subject to copyright under 17 USC 105 and is also made available for use under a CC0 license.

Supplementary materials

Figure S1. Touch training protocol and pretraining performance

A. Schematic illustration of how animals were trained to touch the screen. The intertrial interval (ITI) was initiated after the reward was collected but the duration of the ITI varied from trial to trial since animals returned to their homecage before initiating the next trial. After the ITI, a blue rectangle was presented which occupied 100% of the screen. A touch anywhere on the screen resulted in a 0.2ml reward and a 2kHz sound for 0.5 sec. Over successive days, the size of the blue rectangle was gradually reduced to 75% and then 50% so that it was centrally located on the screen to hone the animal's dexterity. The touch sensitive area was the same size as the rectangle. **B.** Graph shows the number of trials committed by animals on average (± S.E.M.) across training days when the rectangle occupied 100%, 75%, and 50% of the touch sensitive space. **C.** Mean (± S.E.M.) response latency training days. Median values were used since animals voluntarily chose to enter the test box within the 2 hr training session which varied the mean from trial to trial. **D.** Graph shows reward collection latency across training days.

Figure S1

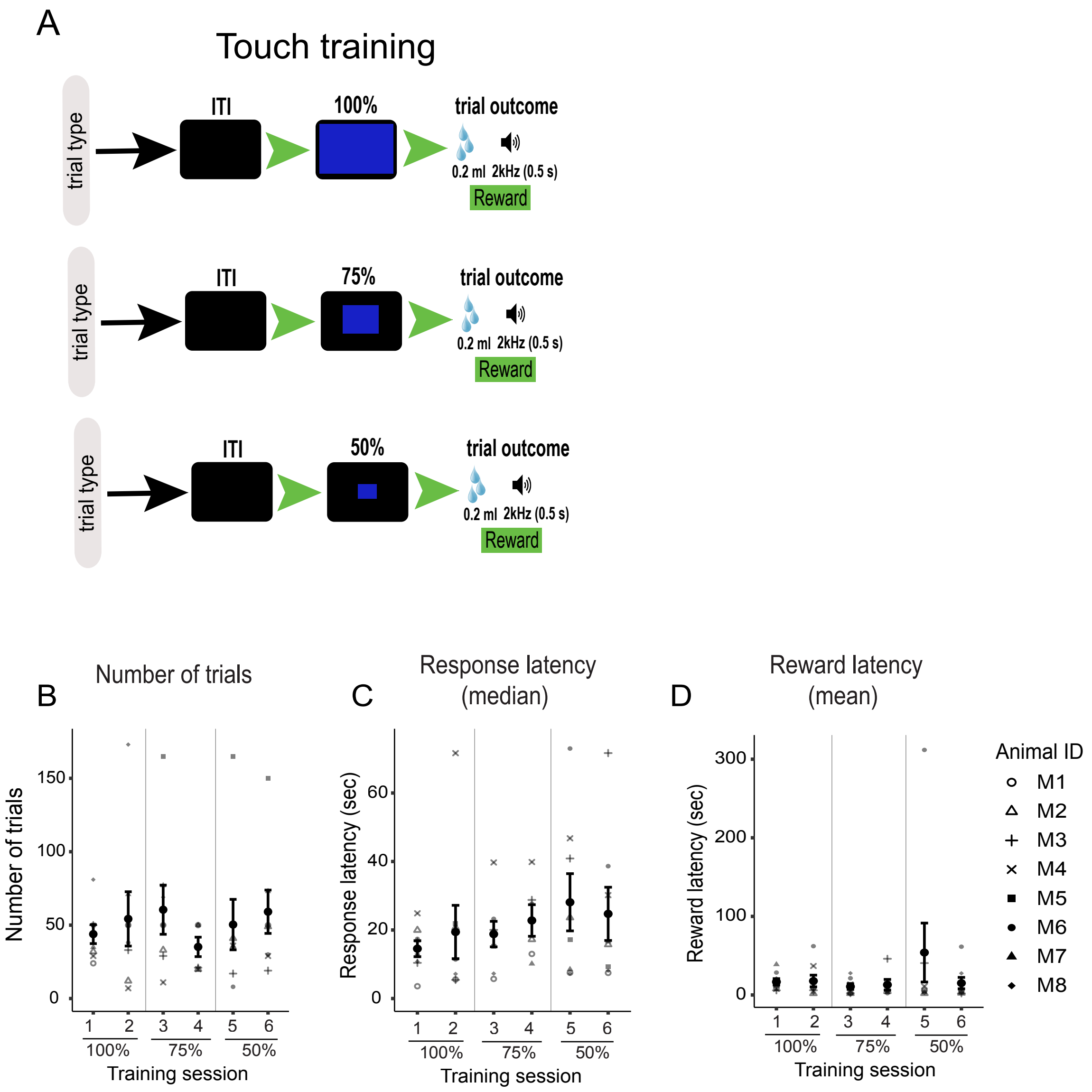
A
Touch training
trial type
ITI
100%
trial outcome
0.2 ml 2kHz (0.5 s)
Reward
trial type
ITI
75%
trial outcome
0.2 ml 2kHz (0.5 s)
Reward
trial type
ITI
50%
trial outcome
0.2 ml 2kHz (0.5 s)
Reward
B
Number of trials
Number of trials
0
50
100
150
1 2 3 4 5 6
100% 75% 50%
Training session
C
Response latency (median)
Response latency (sec)
0
20
40
60
1 2 3 4 5 6
100% 75% 50%
Training session
D
Reward latency (mean)
Reward latency (sec)
0
100
200
300
1 2 3 4 5 6
100% 75% 50%
Training session
Animal ID
M1
M2
M3
M4
M5
M6
M7
M8
